# Novel MSX1 frameshift mutation in a Japanese family with nonsyndromic oligodontia

**DOI:** 10.1038/s41439-021-00161-x

**Published:** 2021-07-20

**Authors:** Junya Adachi, Yoshihiko Aoki, Tadashi Tatematsu, Hiroki Goto, Atsuo Nakayama, Takeshi Nishiyama, Katsu Takahashi, Masatoshi Sana, Akiko Ota, Junichiro Machida, Toru Nagao, Yoshihito Tokita

**Affiliations:** 1 Department of Maxillofacial Surgery, School of Dentistry, Aichi-Gakuin University, Nagoya, Japan; 2 Department of Disease Model, Institute for Developmental Research, Aichi Developmental Disability Center, Kasugai, Japan; 3 Department of Cellular Pathology, Institute for Developmental Research, Aichi Developmental Disability Center, Kasugai, Japan; 4 Department of Public Health, School of Medicine, Nagoya City University, Nagoya, Japan; 5 Dentistry & Oral Surgery,Tazuke Kofukai, Medical Research Institute, Kitano Hospital, Osaka, Japan; 6 Nagoya Orthodontic Clinic, Nagoya, Japan; 7 Department of Oncology, Toyota Memorial Hospital, Toyota, Japan; 8 Department of Oral and Maxillofacial Surgery, Toyota Memorial Hospital, Toyota, Japan

**Keywords:** Diseases, Mutation

## Abstract

Congenital tooth agenesis is a common anomaly in humans. We investigated the etiology of human tooth agenesis by exome analysis in Japanese patients, and found a previously undescribed heterozygous deletion (NM_002448.3(MSX1_v001):c.433_449del) in the first exon of the *MSX1* gene. The deletion leads to a frameshift and generates a premature termination codon. The truncated form of MSX1, namely, p.(Trp145Leufs*24) lacks the homeodomain, which is crucial for transcription factor function.

Congenital tooth agenesis is a common human anomaly classified into two subtypes based on the number of permanently missing teeth, excluding the third molars. Hypodontia is a condition defined as the absence of 1–5 permanent teeth (OMIM: #106600, #604625); oligodontia is another type of tooth agenesis in which six or more teeth are absent (OMIM: #167416). In most countries, ~5–10% of the total population is affected by congenital tooth agenesis, excluding the third molars^1,2^. Although oligodontia morbidity is more rarely observed, i.e., in 0.08–0.16% of the population, it shows high heritability^3^. A series of genetic studies have revealed the causative genes of human tooth agenesis, which include muscle segment homeobox1 (*MSX1*), paired box 9 (*PAX9*), ectodysplasin A (*EDA*), ectodysplasin A receptor (*EDAR*), and EDAR-associated death domain (*EDARADD*). In addition, several genes of the WNT/beta-Catenin signaling cascade, such as wingless-type MMTV integration site 10 A (*WNT10A*), low-density lipoprotein receptor-related protein 6 (*LRP6*), and axis inhibition protein 2 (*AXIN2*), are associated with human tooth malformation. These proteins play pivotal roles during early human development, including odontogenesis; thus, a mutation in one of the genes may cause both nonsyndromic and syndromic tooth agenesis^4–9^. MSX1 and PAX9 are homeoprotein transcription factors expressed in the dental mesenchyme with important roles in expression of mesenchymal bone morphogenetic protein 4 (*BMP4*), which promotes dental development^10,11^.

Here, we analyze Japanese patients with oligodontia diagnosed on the basis of clinical and radiographic examinations. Saliva samples were obtained from patients after they provided informed consent to participate in the study, which was approved by the Institutional Review Board of Aichi-Gakuin University, TOYOTA Memorial Hospital, and the Institute for Developmental Research, Aichi Developmental Disability Center. In the studied family, the proband (II-1) and her father (I-1) showed symptoms, i.e., loss of eight and six teeth, respectively (Fig. 1A–C). Nevertheless, we did not analyze the nucleotide sequence of the *MSX1* gene of the father because of disagreement with informed consent. Orofacial cleft, craniofacial abnormalities, or other health problems, including those related to ectodermal organs, such as the hair, nails, and sweat glands, were not noted in any of the affected family members.

**Fig. 1:**
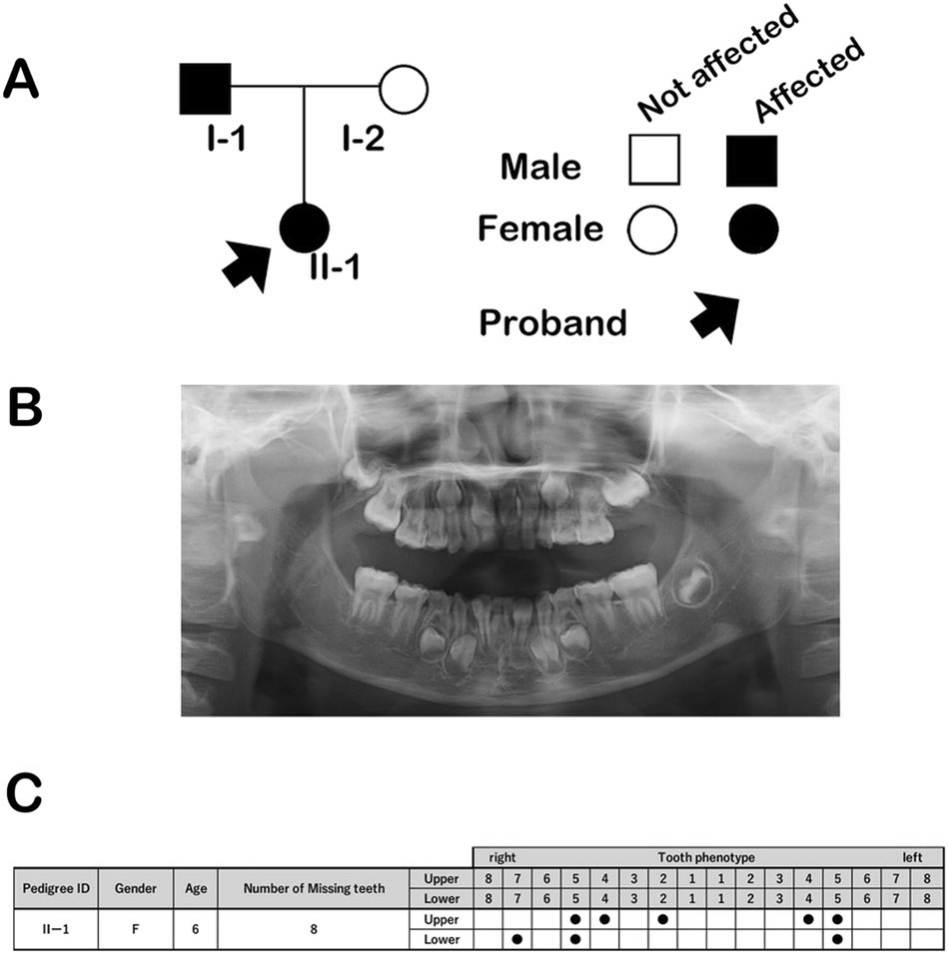
Pedigree of the case patients and missing teeth patterns. **A** The pedigree of the family with familial tooth agenesis. Squares and circles indicate male and female family members, respectively. Filled arrow indicates the proband. Black symbols indicate individuals who were clinically diagnosed with isolated tooth agenesis. **B** Panoramic tomogram of the proband, patient II-1. **C** Missing tooth pattern of the proband (II-1).

Genomic DNA was extracted from saliva using Oragene DNA (OG-500; DNA Genotek, Ontario, Canada) according to the manufacturer’s instructions. Briefly, each saliva sample was mixed with prepIT L2P (PT-2LP; DNA Genotek), incubated on ice, and centrifuged for 5 min at 15,000 × *g*. The supernatant was mixed with EtOH to precipitate DNA. After centrifugation, the DNA pellet was dissolved in elution buffer. Mutational analysis by whole-exome sequencing was subsequently performed according to our previously reported method^12^.

Whole-exome sequencing identified a novel, heterozygous, 17-base pair (TGGATGCAGAGCCCCCG) deletion in the first exon of the *MSX1* gene (NM_002448.3(MSX1_v001):c.433_449del; c.1 is the A of the ATG translation initiation codon of the *MSX1* mRNA; NM_002448.3 in the GenBank database; Fig. 2A). This mutation was not present in the following online databases: dbSNP, 1000 Genomes, and NHLBI exome variant project (http://evs.gs.washington.edu/EVS/). The deletion results in an amino acid substitution of the 145th tryptophan (NP_002439.2) to leucine, and the stop codon following an unrelated peptide sequence consists of 23 amino acid residues (NM_002448.3(MSX1_i001):p.(Trp145Leufs*24); Fig. 2B). Whole-exome sequencing data for the DNA samples did not involve other known causative genes for nonsyndromic tooth agenesis, e.g., *PAX9*, *WNT10A*, *LRP6*, *EDA1*, *PITX2*, *AXIN2*, *EDA1*, *EDAR*, and *EDARADD*.

**Fig. 2:**
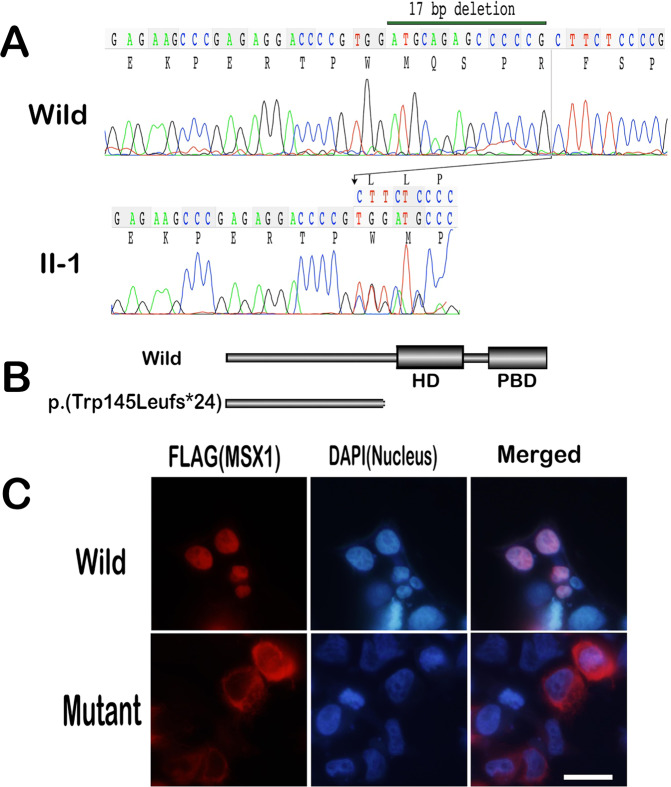
Nucleotide substitution and immunolocalization of mutant MSX1. **A** Sequencing results for the MSX1 gene in the control (upper panel). Heterologous peaks of the nucleotide sequence for tooth agenesis were detected in the patient’s *MSX1* gene (lower panel). **B** Schematic diagram of wild-type MSX1 and the mutant; p.(Trp145Leufs*24). The MSX1 mutant is a C-terminus-truncated form lacking the homeodomain/MH4 and the PIAS-binding domain. PBD PIAS-binding domain (amino acids 265–297). **C** Nuclear localization of wild-type and truncated MSX1 in HEK293 cells. FLAG-tagged wild-type MSX1 immunolocalized to the nucleus of transfected HEK293 cells, but immunoreactivity of the mutant MSX1 was diffusely detected in the cytoplasm. MSX1 (FLAG, red); nuclei [4′,6-diamidino-2-phenylindole (DAPI), blue]. Bar = 10 µm.

The 17-bp deletion generates an MSX1 protein with a C-terminus lacking the homeodomain. It has previously been demonstrated that the homeodomain (amino acids 175–229) plays a pivotal role in molecular interactions with DNA^13^ and other transcription factors related to tooth development, such as PAX9, TATA-binding protein, and DLX family members^14^. The homeodomain is also associated with nuclear transport^15^, which is crucial for the biological function of transcription factors. We expressed mutant MSX1 in HEK293 cells to confirm the nuclear transport defect. Although immunoreactivity of wild-type MSX1 was detected in nuclei, as previously reported^16^, truncated MSX1 was mainly found in the cytoplasm (Fig. 2C).

In addition to its nonsyndromic form, haploinsufficiency of *MSX1* causes the syndromic form of tooth agenesis that includes cleft lip and/or palate^17,18^. To date, 50 *MSX1* gene mutations, including four truncated variants, have been identified in patients with syndromic and nonsyndromic tooth agenesis. Although amino acid substitutions in patients with tooth agenesis cluster in the homeodomain (70%), no variants associated with cleft lip and/or palate without tooth malformation have been identified in it^19^. Previous biochemical analyses have shown that some single-amino acid substitutions in the homeodomain of MSX1 can affect the transcriptional suppression activity of the *MyoD* promoter, which is one of the targets of MSX1^20^. Because MSX1 strongly suppresses target gene expression and represses cell differentiation both in vitro and in vivo^21^, it sustains cellular proliferation in the tooth germ during odontogenesis for robust tooth development in terms of size, number, and shape.

In summary, previous reports and the current results indicate that the p.(Trp145Leufs*24) mutation impairs the molecular function of MSX1; thus, the identified nucleotide deletion is the cause of the tooth agenesis in the studied family. Because of the pathogenic relationship between particular *MSX1* mutations and facial clefts, we believe that clarifying *MSX1* gene variations will help to improve the precision of genetic counseling to patients with odontogenic malformations and/or facial clefts.

## HGV Database

The relevant data from this Data Report are hosted at the Human Genome Variation Database at 10.6084/m9.figshare.hgv.3039
